# Glutathione metabolism–associated resistance to cisplatin and 5-FU in esophageal cancer: a paired transcriptomic study

**DOI:** 10.1186/s12876-026-04664-1

**Published:** 2026-02-10

**Authors:** Masataka Hirasaki, Yutaka Miyawaki, Yasuo Kamakura, Tomonori Kawasaki, Satoshi Yamasaki, Hisayo Fukushima, Yoshinori Makino, Hiroshi Sato, Tetsuya Hamaguchi

**Affiliations:** 1 Department of Clinical Cancer Genomics, Saitama Medical University International Medical Center, 1397-1 Yamane, Hidaka, Saitama 350- 1298 Japan; 2 Department of Gastroenterological Surgery, Saitama Medical University International Medical Center, 1397-1 Yamane, Hidaka, Saitama 350- 1298 Japan; 3 Department of Pathology, Saitama Medical University International Medical Center, 1397-1 Yamane, Hidaka, Saitama 350-1298 Japan; 4 Department of Medical Oncology, Gastroenterological Oncology, Saitama Medical University International Medical Center, 1397-1 Yamane, Hidaka, Saitama 350-1298 Japan

**Keywords:** Esophageal cancer, Neoadjuvant chemotherapy, RNA sequence, Cisplatin plus 5-FU (CF)

## Abstract

**Background:**

Cisplatin plus 5-fluorouracil (CF) remains a cornerstone of treatment for locally-advanced esophageal cancer (EC). However, resistance to CF-based neoadjuvant chemotherapy (NAC) remains a major clinical challenge. Clarifying whether resistance-associated molecular features are induced by treatment or already present before therapy is crucial for improving patient stratification.

**Methods:**

RNA sequencing was performed on paired tumor specimens from patients with EC treated with CF-based NAC, including pre-treatment biopsies (PreNAC) and post-treatment surgical samples (PostNAC) (*n* = 15; recurrence, *n* = 7; non-recurrence, *n* = 8). Differential gene expression analysis was conducted across four groups defined by sample timing (Pre vs. PostNAC) and recurrence status.

**Results:**

PostNAC transcriptional changes in recurrent tumors were relatively limited, with only 53 genes upregulated and 54 genes downregulated compared with PreNAC samples. Notably, genes involved in glutathione metabolism emerged as the dominant resistance-associated pathway and were consistently enriched in recurrent tumors at both PreNAC and PostNAC stages. Key glutathione-related genes, including GSTP1 and GPX2, were highly expressed in recurrent cases, indicating that major resistance-associated molecular features are often already established prior to chemotherapy rather than being newly acquired during NAC.

**Conclusions:**

Our findings suggest that the molecular characteristics associated with CF resistance are frequently present before the initiation of chemotherapy. These results highlight the clinical potential of pretreatment molecular profiling as a strategy to guide therapeutic decision-making and optimize CF-based NAC in EC.

**Supplementary Information:**

The online version contains supplementary material available at 10.1186/s12876-026-04664-1.

## Background

 Esophageal cancer (EC) remains a major global health burden, ranking seventh in incidence and sixth in cancer-related mortality worldwide [1]. In Japan, the histological type of esophageal cancer remains predominantly squamous cell carcinoma, and neoadjuvant chemotherapy (NAC) with cisplatin plus 5-fluorouracil (CF) followed by surgery has long been the standard treatment for stage II–III Esophageal squamous cell carcinoma (ESCC) [2]. Although more intensive regimens, such as docetaxel plus CF (DCF), have demonstrated improved survival [3, 4], CF-based therapy continues to play a central role in EC treatment, including in combination with immune checkpoint inhibitors for advanced or recurrent disease [5–7]. However, resistance to CF-based chemotherapy remains a critical clinical challenge, underscoring the need to elucidate its underlying molecular mechanisms.

Multiple mechanisms of chemotherapy resistance have been proposed in EC, including enhanced DNA repair capacity, metabolic adaptation, and stress-response pathways. Autophagy and mitophagy have also been implicated as potential contributors to therapeutic resistance, with increased expression of mitophagy-related proteins such as PINK1 reported to be associated with poor response to NAC [8–10]. Moreover, studies using paired tumor organoids derived from before and after NAC have suggested that chemotherapy can induce adaptive transcriptional programs related to epithelial–mesenchymal transition (EMT) and hypoxia, supporting the concept of acquired resistance under therapeutic pressure [11]. Despite these advances, a fundamental question remains unresolved: to what extent is chemotherapy resistance in EC driven by molecular features that are already present at diagnosis versus those that are acquired or enriched under CF-based NAC? While both pre-existing and therapy-induced resistance mechanisms have been suggested, their relative contributions in clinical EC remain unclear, largely due to the limited availability of paired pre- and post-treatment tumor samples.

In this study, we performed comprehensive transcriptomic profiling of paired tumor specimens obtained before (PreNAC) and after (PostNAC) CF-based NAC in patients with EC. This study was originally designed based on the assumption that resistance-associated molecular features would be predominantly acquired or enriched under the selective pressure of chemotherapy. By comparing gene expression patterns between recurrent and non-recurrent cases across these two time points, we sought to determine whether key resistance-related molecular programs are primarily induced during NAC or are already detectable prior to treatment. Through this approach, we aimed to clarify the timing and nature of CF resistance–associated molecular features and to explore the potential clinical value of pretreatment molecular profiling for optimizing NAC strategies in EC.

## Methods

### Tissue samples

This study was approved by the Institutional Review Board of Saitama Medical University International Medical Center (approval numbers: 2022 − 113 and 2024-055), and the requirement for informed patient consent was waived owing to the retrospective nature of the study.

Ninety-one patients with EC who underwent neoadjuvant CF regimen plus radical surgery at Saitama Medical University International Medical Center between May 2012 and June 2020 were enrolled. Of these, 76 patient samples with complete biopsy and surgical specimens were included in the analyses (Table 1). As biopsy specimens have been analyzed in previous studies, this study analyzed surgical specimens after NAC [12]. The tissue specimens were processed, embedded in paraffin blocks, and used for further analysis. Tumor cells in the tissue specimens were visually and microscopically determined by a pathologist using hematoxylin and eosin (H&E) stained sections.

Continuous variables are presented as median and range and were compared between the non-recurrence and recurrence groups using the Mann–Whitney U test. Categorical variables are presented as number and percentage. Comparisons of categorical variables were performed using Fisher’s exact test when expected cell counts were small, or the chi-square (χ²) test when appropriate.

### RNA extraction and library preparation for RNA sequencing

Total RNA was isolated from formalin-fixed paraffin-embedded (FFPE) PostNAC surgical specimens (*n* = 15) from the same patients whose PreNAC biopsy RNA-sequencing (RNA-seq) data had been analyzed in a previous study [12], enabling direct paired transcriptomic comparisons (Additional File 1). RNA quality was assessed using DV200 values as described previously [13], and only samples with DV200 ≥ 35 were included for RNA-seq analysis. This quality threshold was applied uniformly to both PreNAC biopsy and PostNAC surgical FFPE specimens. Libraries for RNA-seq were prepared from total RNA as previously described [13].

### Data analysis for RNA sequences

Data analysis followed previously reported methods with some modifications [13]. For RNA-seq analyses, samples were classified into four groups according to treatment timing and recurrence status: PreNAC recurrence, PreNAC non-recurrence, PostNAC recurrence, and PostNAC non-recurrence. Differential gene expression analyses were performed by comparing recurrence versus non-recurrence groups at each time point, as well as by comparing PreNAC and PostNAC samples within the same recurrence category, in accordance with the presentation of results. The significance estimate of the differences in gene expression, such as the p-value and false discovery rate (FDR), was calculated from the expected counts of RNA-Seq by Expectation-Maximization using the edgeR package in R (https://bioconductor.org/packages/release/bioc/html/edgeR. html). Differentially expressed genes (DEGs) were defined as genes that showed a two-fold or greater difference in the expression level of transcripts per million (TPM) between the recurrence and non-recurrence groups, with a significant difference of *p* < 0.05. The TPM values of the 38 genes defined as DEGs based on FDR criteria (DEGs_FDR) were normalized using Z-scores, and principal component analysis (PCA) and heat maps were generated using the R package. Statistical thresholds were selected according to the analytical objective, with *p* < 0.05 used for exploratory analyses and FDR-based criteria applied for defining representative gene sets in downstream analyses. The volcano plot was generated using GraphPad Prism 10.0. for Mac (GraphPad Software). STRING analysis (https://string-db.org/) was used to identify protein–protein interaction (PPI) networks associated with highly expressed transcripts.

### Gene ontology and pathway analysis

To characterize the molecular and functional aspects of the DEGs, Gene Ontology (GO) and Kyoto Encyclopedia of Genes and Genomes (KEGG) pathway analyses were performed using the online DAVID database (https://david.ncifcrf.gov/).

### Gene set enrichment analysis

Using trimmed means of M values, which are normalized count data, gene set enrichment analysis (GSEA) was performed according to the methods described on the GSEA website (http://www.gsea-msigdb.org/gsea/index.jsp).

### Multiplex expression analysis

Multiplex expression analysis was performed using RNAseqChef (https://imeg-ku.shinyapps.io/RNAseqChef/) [14] to complement standard differential expression analysis by characterizing coordinated gene expression dynamics across treatment timing and recurrence status. Raw counts data were used, and cut-off conditions were set for the difference between BiopsyRecurrence and SurgicalRecurrence with fold change (FC) ≥ 2 and FDR ≤ 0.25.

### Target sequencing in our clinical esophageal squamous cell carcinoma cases

Fifty-six autophagy- and esophageal squamous cell carcinoma (ESCC)-related genes were selected for targeted-enrichment sequencing. Details of DNA extraction, library preparation, and data analysis for target capture sequencing have been previously described [12]. Single-nucleotide variant (SNV) and small insertion/deletion (INDEL) identification was based on Mutect2 of the Genome Analysis Toolkit, the detected variants were annotated using ANNOVAR, and pathogenicity was assessed using the ClinVar_20210501 database [15, 16]. SNVs with multiple allelic sites were excluded from analysis. The OncoPrint plot was visualized using R software. A 2 × 2 cross-tabulation table was created with and without variants, and with and without recurrence. Based on the cross-tabulation table, Fisher’s exact test was performed using R to examine the association between gene variants and recurrence.

## Results

### Global transcriptomic differences between recurrent and non-recurrent tumors after CF-based NAC

Patient characteristics of the recurrence and non-recurrence groups are summarized in Table 1. To elucidate the biological characteristics of patients with poor response to CF regimens, RNA-seq was performed on paired tumor specimens (PreNAC biopsies and PostNAC surgical specimens) collected from patients with EC who received CF-based NAC. As RNA-seq results for PreNAC specimens have been previously reported, this study performed a comprehensive gene expression analysis using total RNA extracted from PostNAC FFPE surgical specimens [12].

**Table 1:** Clinical characteristics of the patients included in this study

	Non-recurrence (*n* = 34)	Recurrence (*n* = 42)		Test of significance	*p* value
Age years					
Median (range)	68 (51–79)	69 (55–80)		U	*p* = 0.187
Gender (%)					
Male / Female	31 (91%) / 3 (9%)	38 (90%) / 4 (10%)		Fisher’s exact test	*p* = 1.000
Organization type (%)					
Basaloid/SCC	1 (3%) / 33 (97%)	2 (5%) / 40 (95%)		Fisher’s exact test	*p* = 1.000
Neo-adjuvant course (%)					
1 / 2	5 (15%) / 29 (85%)	5 (12%) / 37 (88%)		Fisher’s exact test	*p* = 1.000
Tumor location (%)					
Upper / Middle / Lower	2 (6%) / 17 (50%) / 15 (44%)	9 (21%) / 15 (36%) / 18 (43%)		χ²	*p* = 0.024
cT category (%)					
cT1 / T2 / T3	0 (0%) / 0 (0%) / 34 (100%)	1 (2%) / 2 (4%) / 39 (94%)		Fisher’s exact test	*p* = 0.248
cN category (%)					
cN0 / N1 / N2	15 (44%) / 10 (29%) / 9 (27%)	12 (29%) / 21 (50%) / 9 (21%)		χ²	*p* = 0.180
cM category (%)					
cM0 / M1	33 (97%) / 1 (3%)	40 (95%) / 2 (5%)		Fisher’s exact test	*p* = 1.000
cStage (%)					
I / II / III/ IV	0 (0%) / 15 (44%) / 18 (53%) / 1 (3%)	1 (2%) / 12 (30%) / 27 (64%) / 2 (4%)		Fisher’s exact test	*p* = 0.49

*Abbreviations*: *n* number of patients, *SCC* squamous cell carcinoma, *cT* clinical tumor depth category, *cN* clinical lymph node metastases category, *cM* clinical distant metastases category, *cStage* clinical stage based on the TNM classification, *Neo-adjuvant* course number of neoadjuvant chemotherapy cycles, *U* Mann–Whitney U test, *χ²* chi-square test.

**Fig. 1:**
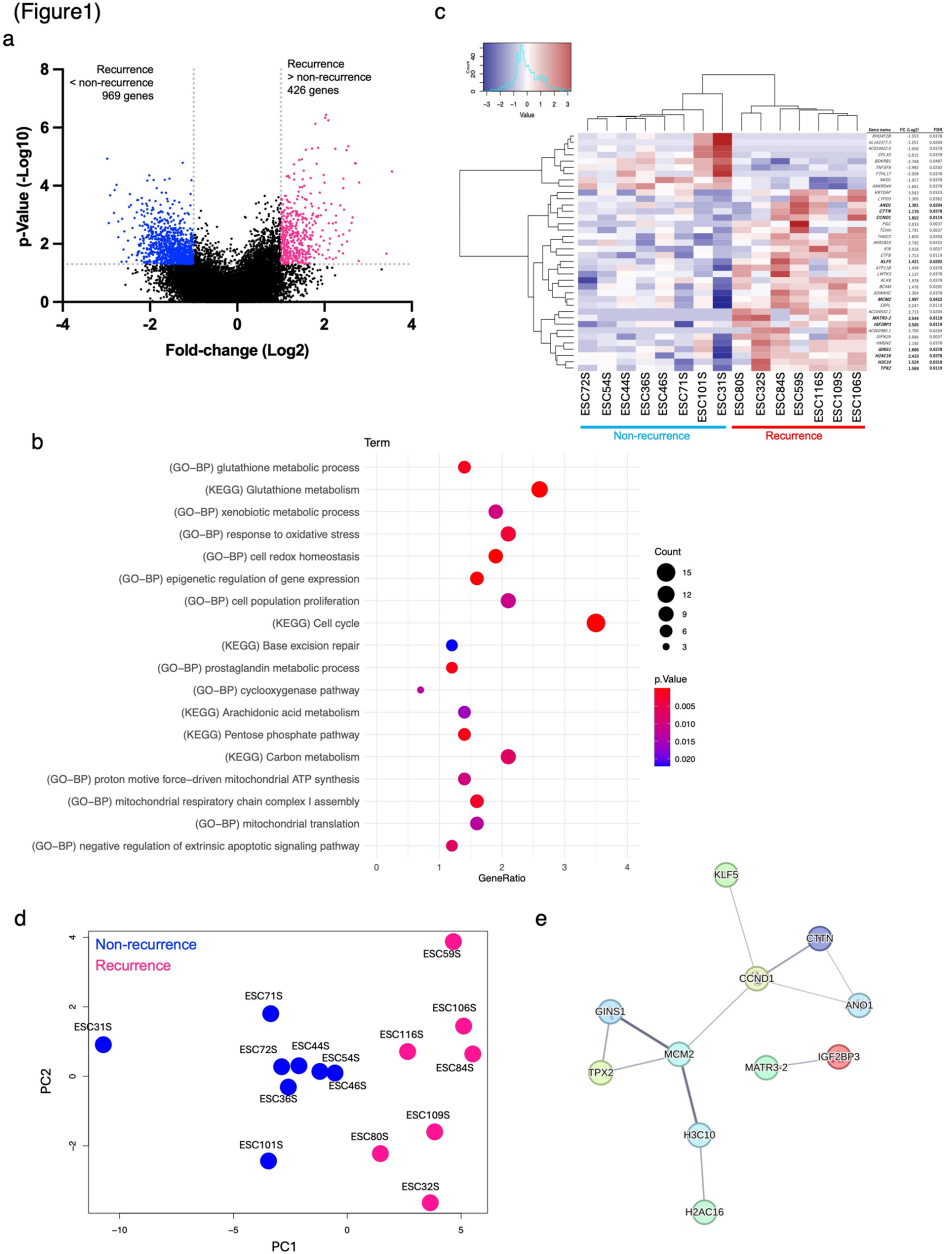
RNAseq of esophageal cancer specimens after neoadjuvant chemotherapy. (**a**) Volcano plot of DEGs between recurrence (*n* = 7) and non-recurrence (*n* = 8) groups. (**b**) Summary of biological processes in gene ontology (GO-BP) and KEGG-pathway analysis of genes with elevated expression in recurrence. (**c-d**) TPM values of 38 genes defined as DEGs_FDR were normalized using Z-scores, (**c**) Heatmap, and (**d**) PCA was performed. (**e**) STRING network analysis of 38 genes defined as DEGs_FDR. DEG, differentially expressed genes; KEGG, Kyoto Encyclopedia of Genes and Genomes; PCA, principal component analysis

We analyzed the gene expression differences between the recurrence (*n* = 7) and non-recurrence (*n* = 8) groups in PostNAC specimens (Additional File 1). As shown in Fig. 1a, comparison of 19,972 protein-coding genes identified a large number of DEGs between recurrent and non-recurrent tumors, highlighting substantial transcriptomic differences associated with recurrence. Specifically, 426 and 969 genes showed significantly higher and lower expression, respectively, in the recurrence group than in the non-recurrence group, based on the criteria of FC ≥ 2 and *p* ≤ 0.05 (Additional Files 2–3).

### Pathway-level analyses identify glutathione metabolism as a dominant signature associated with recurrence

Functional annotation analysis revealed enrichment of oxidative stress–related pathways, including glutathione (GSH) metabolism, in recurrent tumors (Fig. 1b), suggesting enhanced redox adaptive capacity in this group. The results showed that at both PreNAC and PostNAC stages, the recurrent group exhibited significant enrichment of processes related to oxidative stress resistance, including oxidative stress response and GSH metabolism. Furthermore, these also included pathways supporting energy supply in tumor cells, such as metabolic pathways and mitochondrial ATP synthesis, as well as molecules involved in DNA repair systems, including base excision repair (Fig. 1b, Additional Files 4‒6).

**Fig. 2:**
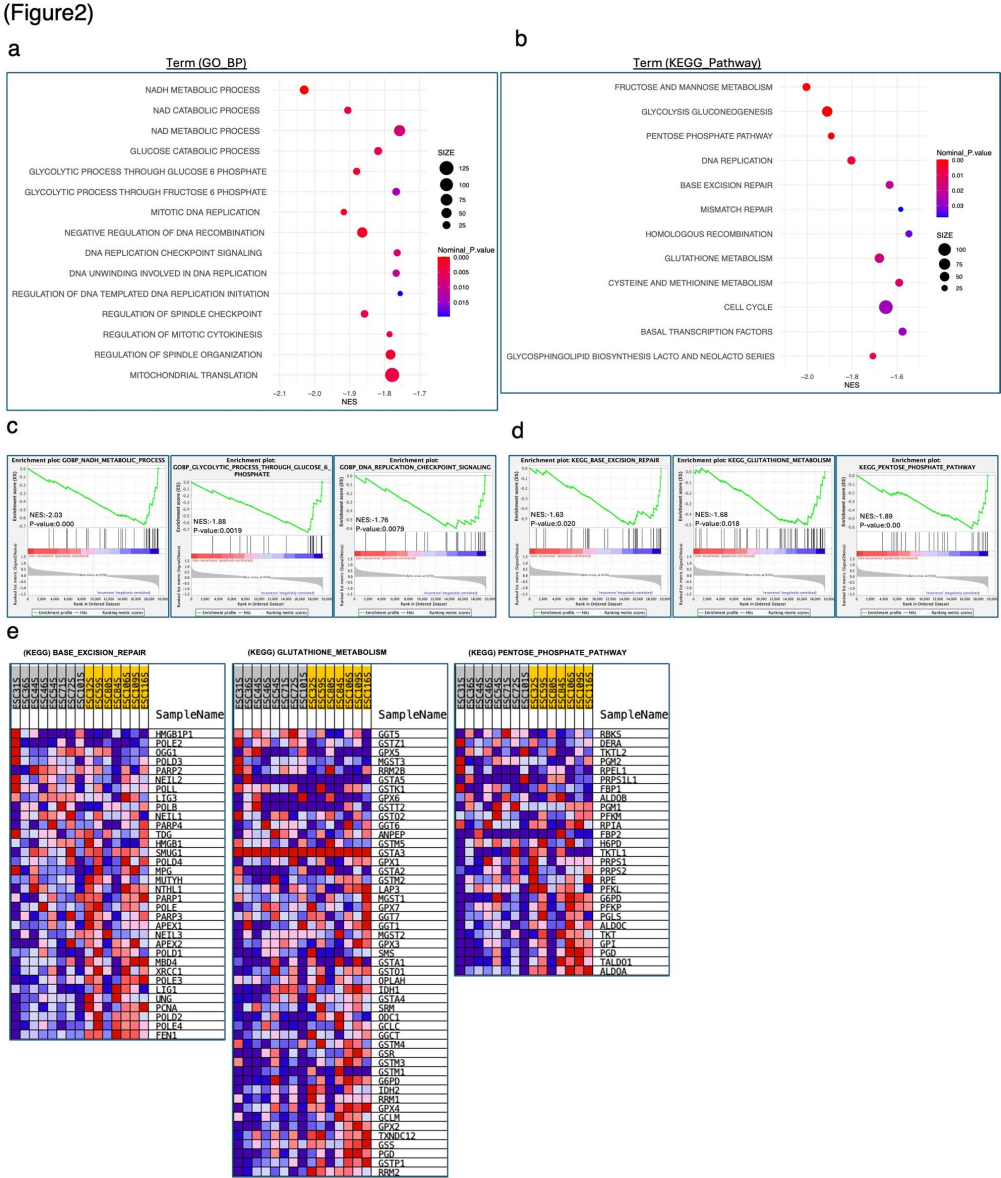
GSEA based on expression analysis of esophageal cancer samples after neoadjuvant chemotherapy. **a-b** Summary of GO and KEGG analysis of enriched genes in the recurrence group by GSEA. **c** GSEA plots of the GO-BP enriched in the recurrence group. **d** GSEA plots of the KEGG pathway enriched in the recurrence group. **e** Heatmap of “BASE_EXCISION_REPAIR” and “GLUTATHIONE_METABOLISM”, “PENTOSE_PHOSPHATE_PATHWAY” from GSEA. GO, gene ontology; KEGG, Kyoto Encyclopedia of Genes and Genomes; GSEA, gene set enrichment analysis; NES, normalized enrichment score

GSEA was performed to elucidate the molecular mechanisms underlying the recurrence groups. The results showed that biochemical processes related to energy metabolism, such as NADH metabolism and glycolysis through glucose-6-phosphate, as well as DNA replication checkpoint signaling involved in DNA replication and repair, were significantly enriched at both the PreNAC and PostNAC time points (Fig. 2a, Additional Files 7a, 8, and 9). Furthermore, KEGG pathway analysis revealed significant enrichment of gene expression in pathways such as GSH metabolism, pentose phosphate pathway, and DNA replication (Fig. 2b–e, Additional File 7b). These findings suggest that recurrent tumors exhibit enhanced oxidative stress resistance, metabolic adaptability, and DNA repair capacity.

### Possibility of predicting recurrence by analysis of gene set expression

Differential expression analysis was conducted using thresholds of FC ≥ 2 and FDR ≤ 0.05. Consequently, 38 genes were identified as DEGs based on the FDR criteria (DEGs_FDR). Hierarchical cluster analysis was performed using DEGs_FDR (clustering method: Ward’s method [ward.D2]). Samples from the recurrence and non-recurrence groups were separated into two distinct clusters based on their expression profiles (Fig. 1c). PCA was performed using the same gene expression data. Scatter plots of the first principal component (PC1) clearly separated the recurrence and non-recurrence groups, indicating that PC1 effectively explained the presence or absence of recurrence (Fig. 1d). This result suggests a distinct difference in gene expression between the recurrence and no-recurrence groups.

### PPI network analysis

PPI network analysis was performed on 38 genes identified using the STRING database with an FDR threshold of ≤ 0.05, revealing that 11 of the 38 genes were attributed to the co-expression of genes based on publicly available datasets (Fig. 1e). All 11 genes were highly expressed in the relapse group, characterized by a specific expression profile. Further analysis of the interactions within the PPI network and associated biological processes predicted that these gene clusters were involved in processes that contribute to cancer cell proliferation enhancement (such as cell cycle and proliferation, DNA repair and replication, chromosome structure, and gene expression regulation), suggesting that they may reflect the mechanism of tumor progression in the recurrent group.

### Key GSH metabolism–related genes are consistently upregulated in recurrent tumors

**Fig. 3:**
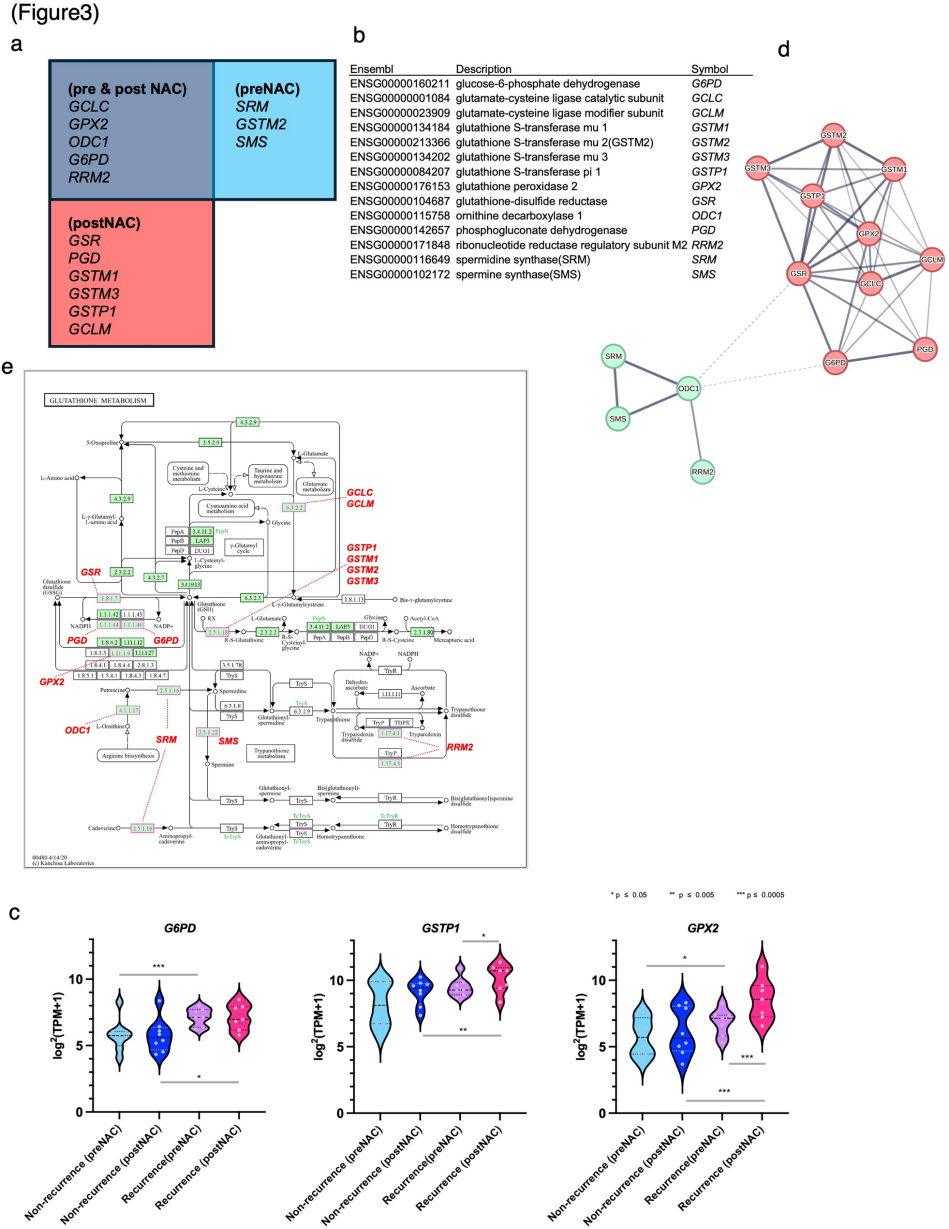
Genes contributing to the glutathione metabolic pathway. (**a-b**) Venn diagram and list of glutathione metabolism-related genes whose expression increased in PreNAC and PostNAC samples in the recurrence group. (**c**) Violin plots showing gene expression levels of glutathione metabolism-related genes GSTP1, G6PD, and GPX2 in PreNAC and PostNAC samples from the recurrence group and non-recurrence group. (**d**) STRING network analysis of 14 glutathione metabolism-related genes whose expression increased in PreNAC and PostNAC samples in the recurrence group. (**e**) Mapping of 14 genes in the KEGG glutathione metabolism pathway. The pathway map was modified based on the KEGG pathway map image (https://www.genome.jp/pathway/mmu04310). KEGG, Kyoto Encyclopedia of Genes and Genomes

In our previous study, we reported that eight genes related to the GSH metabolic pathway were significantly overexpressed in the PreNAC recurrence group. In this study, we confirmed that 11 genes related to the same pathway were significantly overexpressed in the PostNAC recurrence group (Fig. 3a–b). As shown in Fig. 3c, key GSH metabolism–related genes, including GSTP1, GPX2, and G6PD, were upregulated in recurrent tumors, indicating sustained activation of GSH-dependent detoxification pathways. Notably, G6PD showed consistently high expression in the recurrence group at both the PreNAC and PostNAC stages, whereas GSTP1 and GPX2 exhibited increased expression predominantly in PostNAC recurrent tumors. Integrated analysis of these results revealed that 14 genes associated with the “GSH metabolism” pathway were highly expressed in the recurrence group. Specifically, three genes were highly expressed only during PreNAC, six showed elevated expression during PostNAC, and five showed consistently high expression at both stages (Fig. 3a). Figure 3d shows the PPI network for the 14 genes involved in GSH metabolism, suggesting that this group of genes is functionally closely interconnected. Figure 3e shows the results of the visualization of the GSH metabolic pathway, including these genes, using KEGG pathway mapping, suggesting the activation of metabolic pathways involved in NADPH production, along with upregulation of the glutathione S-transferase (GST) family.

### Multiplex expression analysis reveals limited transcriptional changes after NAC

**Fig. 4:**
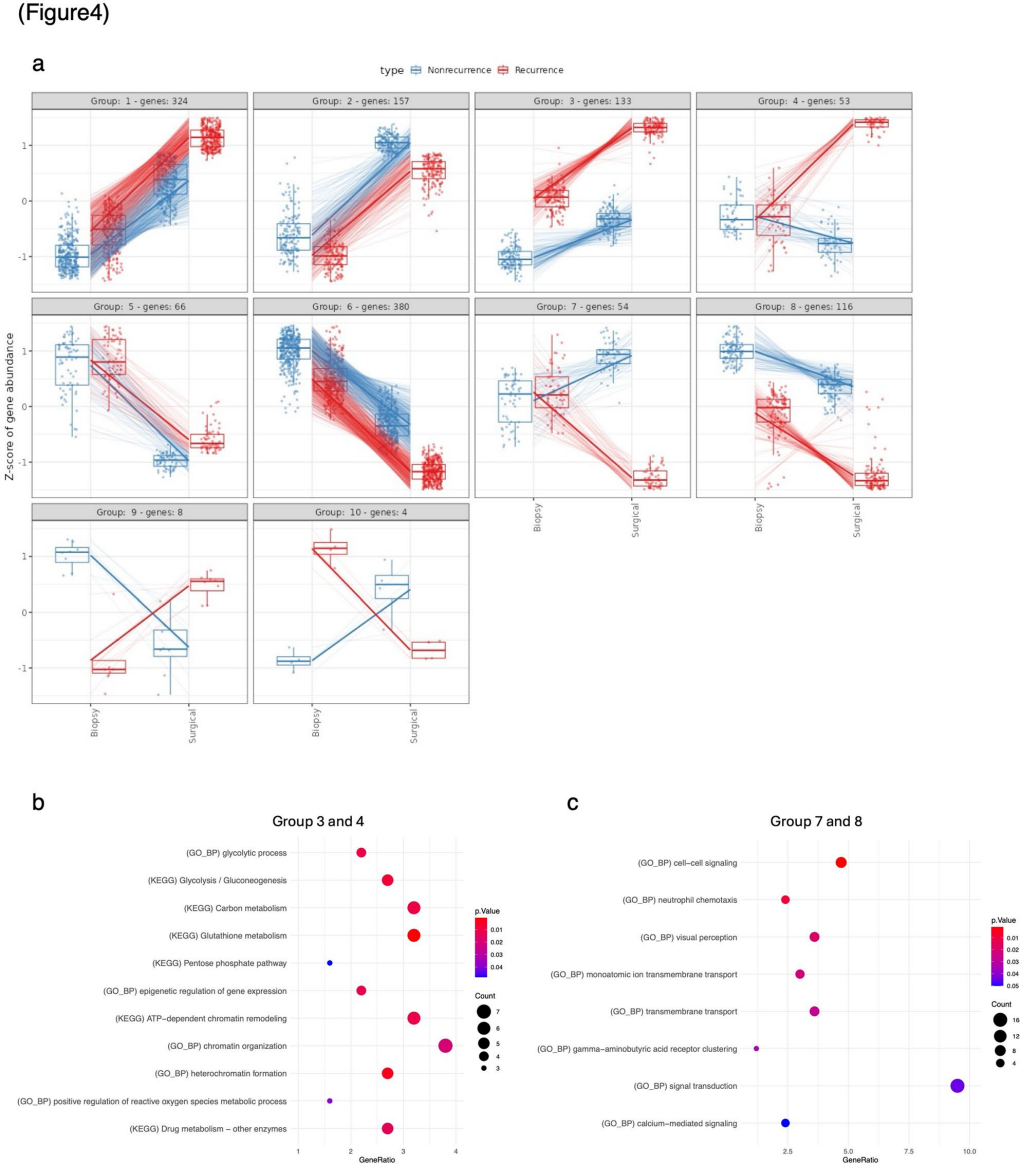
Clustering and expression of DEGs across biopsy and surgical specimens in recurrence and non-recurrence groups. (**a**) Multiple expression analysis was performed using RNAseqChef to identify distinct expression patterns between Biopsy and Surgical specimens in Recurrence (red) and Non-recurrence (blue) groups. Genes were grouped into ten distinct clusters (Group 1 to Group 10) based on their expression dynamics across the two time points. (**b**) Summary of biological processes in gene ontology (GO-BP) and KEGG-pathway (KEGG) analysis of genes with elevated expression in groups 3 and 4. (**c**) Summary of biological processes in gene ontology (GO-BP) analysis of genes with elevated expression in groups 7 and 8. DEG, differentially expressed genes; KEGG, Kyoto Encyclopedia of Genes and Genomes

To clarify the impact of NAC on the therapeutic pressure exerted on tumor tissue, RNAseqChef was used for multiple expression analyses. Biopsy (PreNAC) and surgical (PostNAC) specimens were collected from patients in the recurrence and non-recurrence groups, and gene expression changes before and after treatment were compared. For analysis, the cutoff values for the PreNAC-recurrence vs. PostNAC-recurrence difference were set as FC ≥ 2 and FDR ≤ 0.25. Clustering analysis of the obtained gene expression data using RNAseqChef enabled the classification of the entire gene population into 10 groups based on expression patterns (Fig. 4a, Additional File 10).

We focused particularly on Groups 3 and 4, which showed increased expression in PostNAC samples compared with PreNAC samples in the recurrence group, and conversely on Groups 7 and 8, which showed decreased expression in PostNAC samples. Functional annotation analysis indicated that genes in Groups 3 and 4 were potentially involved in metabolic pathways, such as glycolysis, gluconeogenesis, and carbon metabolism (Fig. 4b–c, Additional Files 11–12). In the recurrence group, under the treatment pressure of NAC, 53 genes (Group 4) were upregulated and 54 genes (Group 7) were downregulated; however, the overall number was limited.

### Genetic alterations are largely stable before and after NAC and are not associated with recurrence

Previous studies performed targeted sequencing of EC and mitophagy-related genes to identify SNVs and INDELs, which were subsequently classified as pathogenic or likely-pathogenic variants by consulting ClinVar. In the PreNAC samples, pathogenic variants were detected in 22 genes, including 17 EC-related genes, in 80 of 82 analyzed samples (97.5%). Furthermore, pathogenic variants in these genes showed no significant correlation with recurrence status [12].

**Fig. 5:**
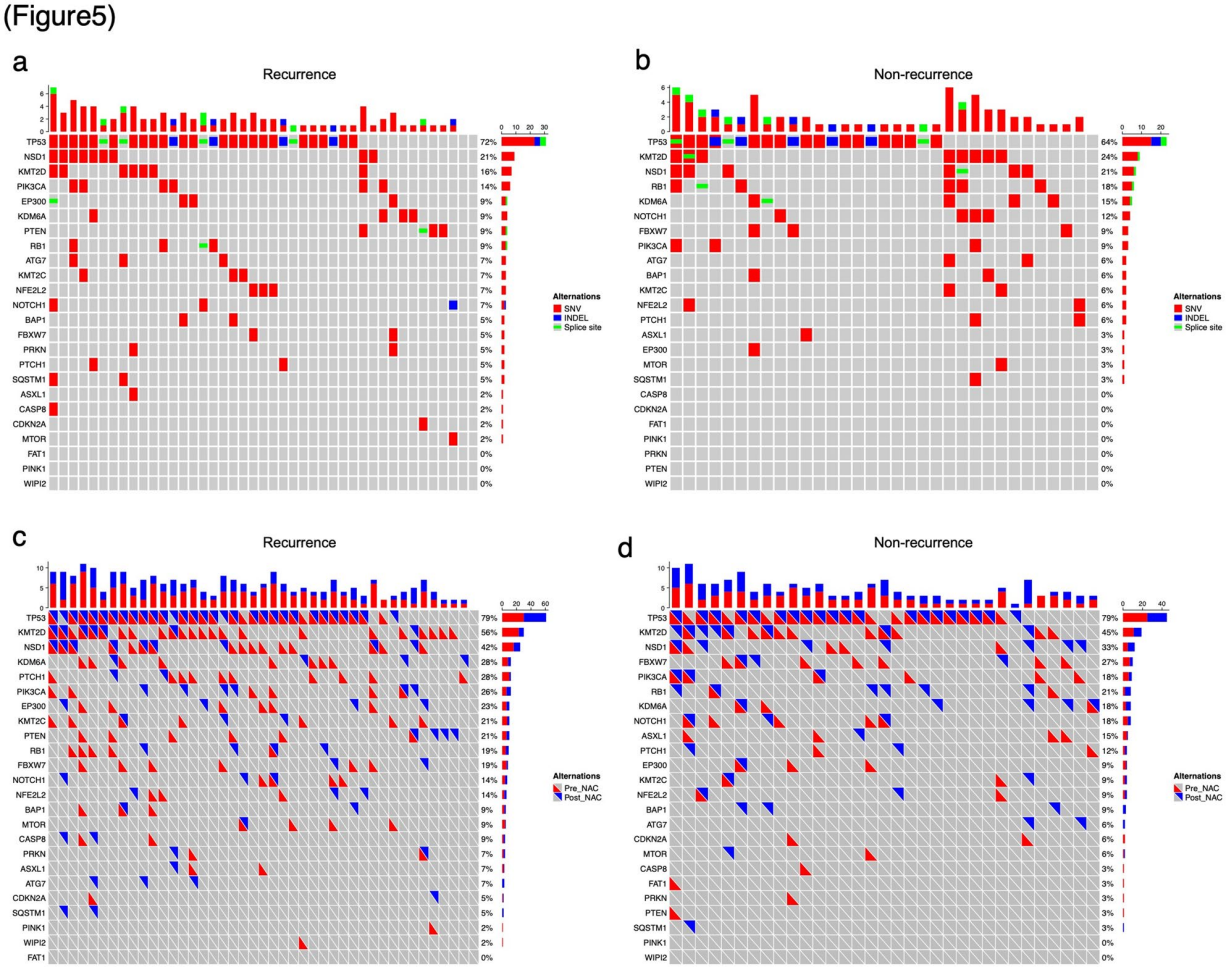
Oncoprints of pathogenic mutations. (**a-b**) Oncoprint visualized the distribution and frequency of pathogenic mutations identified in major cancer-related genes in the recurrence group (**a**) and non-recurrence group (**b**) of PostNAC samples. (**c-d**) Visualized between PreNAC (**c**) and PostNAC (**d**) samples in the recurrence group and non-recurrence group

In this study, using the same gene set, we analyzed pathogenic variants in both PreNAC and PostNAC samples from 76 cases in which both sets of samples were available. At least one pathogenic variant was detected in 73 of the 76 cases (96.1%). Similar to PreNAC, no pathogenic variants showed a significant association with recurrence in PostNAC (Fig. 5a–b, Additional File 13).

Furthermore, evaluation of pathogenic variants pre- and post-NAC revealed the disappearance or emergence of new variants in some cases (Fig. 5c–d). Neither the newly-emerging pathogenic variants nor those that disappeared after NAC showed a significant correlation with recurrence (Additional File 14). These results suggest that variant changes pre- and post-NAC are not associated with recurrence, indicating that the therapeutic pressure from NAC may have only a limited effect on the selection or elimination of specific tumor clones.

## Discussion

Elucidating when and how chemotherapy resistance emerges during CF-based NAC is essential for improving clinical outcomes in EC. Contrary to our initial hypothesis that resistance-associated molecular programs would be broadly acquired under the selective pressure of NAC, we found that treatment-associated transcriptomic changes after NAC were relatively limited, whereas molecular features associated with recurrence—most notably those related to GSH metabolism—were frequently detectable prior to treatment. However, given spatial intratumoral heterogeneity and sampling differences between pretreatment biopsies and post-treatment resection specimens, an alternative interpretation is that these signals may reflect region-specific enrichment of resistant subclones rather than uniform, tumor-wide pre-existence.

In line with this observation, our comprehensive transcriptome analysis of paired tumor specimens obtained before (PreNAC) and after (PostNAC) CF-based NAC revealed that 14 GSH metabolism–related genes were highly expressed in recurrent tumors at the PreNAC stage, the PostNAC stage, or both, supporting the notion that resistance-associated molecular programs are often established prior to chemotherapy rather than being broadly acquired during NAC.

In the present study, GPX2 expression was significantly elevated in the recurrence group and was further increased in PostNAC samples compared with PreNAC samples. GPX2 is a key antioxidant enzyme involved in the detoxification of reactive oxygen species (ROS), which are known mediators of cisplatin- and 5-FU–induced cytotoxicity [17–20]. Consistent with reports in other cancer types showing that GPX2 overexpression promotes cisplatin resistance by suppressing ROS accumulation, our findings suggest that NAC-associated upregulation of GPX2 may enhance intracellular antioxidant defenses, thereby promoting survival of residual tumor cells and contributing to chemoresistance [21, 22].

In this study, multiple GST family genes, including GSTP1, were highly expressed in the recurrence group. GSTP1 has been reported to promote cisplatin detoxification through GSH conjugation, thereby reducing intracellular platinum accumulation, and its high expression has been associated with poor prognosis and chemoresistance in ESCC [23–25]. Consistent with these reports, our findings indicate that not only GSTP1 but also several other GST family members are upregulated in recurrent tumors, suggesting coordinated activation of GSH-dependent drug detoxification pathways. These results support the notion that enhanced GST-mediated conjugation represents a key mechanism contributing to CF resistance in recurrent EC and highlight the potential value of evaluating multiple GST genes rather than a single marker.

Previous studies have suggested that PINK1-dependent mitophagy and chemotherapy-induced transcriptional reprogramming may contribute to acquired resistance after NAC, as exemplified by the association of high PINK1 expression in PostNAC specimens with poor prognosis and by organoid-based analyses showing increased tumorigenicity and EMT/hypoxia-related programs after NAC [10, 11]. Consistent with this concept, we examined treatment-associated transcriptional changes by stratifying samples according to treatment timing and recurrence status. In our analysis, a limited number of genes were specifically altered after NAC in recurrent tumors, with 53 genes upregulated and 54 genes downregulated. Notably, GSTP1 and GSTM3 were included among the upregulated genes, supporting the role of GSH metabolism in resistance. However, the overall magnitude of PostNAC transcriptional change was modest, suggesting that widespread acquisition of new resistance programs during NAC is unlikely. Beyond methodological considerations, this limited PostNAC shift may have biological implications. Resistance after CF-based NAC may be driven predominantly by the selection or enrichment of pre-existing resistant subclones or cell states, rather than by extensive therapy-induced reprogramming. Moreover, clinically-relevant resistance phenotypes may arise through coordinated modulation of a limited number of hub pathways—such as redox regulation, metabolic adaptability, and DNA repair—consistent with the recurrent enrichment of GSH metabolism and replication/repair–related signatures observed in this study.

Targeted sequencing analysis revealed that most pathogenic variants were retained in tumor tissues after NAC, with no significant association between variant retention or emergence and recurrence status. Although some variants appeared or disappeared after treatment, these changes were limited and did not correlate with clinical recurrence. These findings are consistent with previous reports demonstrating the persistence of key driver mutations, such as TP53, after platinum-based NAC and highlighting substantial spatial intratumoral heterogeneity [26]. Given that our analysis was based on single-region sampling before and after NAC, the observed gain or loss of variants is likely influenced by regional sampling differences rather than true clonal elimination or expansion. Overall, the limited impact of NAC on mutational profiles suggests that chemotherapy exerts only modest selective pressure on tumor genomic architecture, and that genetic mutation dynamics alone are unlikely to be the primary drivers of recurrence in EC.

In this study, resistance mechanisms potentially shaped by NAC treatment pressure, including those involving GSH metabolism, were suggested. However, contrary to our initial hypothesis, both transcriptomic and genomic changes induced by NAC were relatively limited. These findings indicate that key molecular features associated with CF resistance are often already present prior to treatment, rather than being broadly acquired during NAC. This study has a few limitations. First, the identified candidate genes and pathways were derived from transcriptomic and integrative bioinformatics analyses, and causal relationships with chemoresistance were not experimentally validated. Second, the relatively small size of the PostNAC RNA-seq cohort may limit statistical power and generalizability, and subtle or heterogeneous tumor cell–specific changes may not have been fully captured by bulk RNA-seq. Furthermore, although clinicopathological characteristics are summarized in Additional File 1, residual confounding by clinical or pathological factors cannot be completely excluded. Moreover, because only a single tumor region per patient was analyzed at each time point, spatial intratumoral heterogeneity may not have been fully captured, which could partially explain the apparent gain or loss of certain variants observed before and after NAC. Future large-scale studies incorporating multi-region sampling and functional validation of GSH pathway–related genes will be required to establish their clinical utility.

## Conclusion

Our findings provide a strong rationale for prospective clinical studies evaluating pretreatment molecular markers to optimize first-line NAC selection, including CF-based regimens, by enabling the identification of resistance-associated programs in EC.

## Supplementary Information

Supplementary Material 1. Additional File 1. Clinical characteristics of patients included in the RNA-seq cohort. Additional File 2. List of differentially expressed genes (DEGs) showing higher expression in the non-recurrence group compared with the recurrence group in PostNAC samples. Additional File 3. List of differentially expressed genes (DEGs) showing higher expression in the recurrence group compared with the non-recurrence group in PostNAC samples. Additional File 5. List of Gene Ontology biological processes (GO-BP) and KEGG pathways enriched among genes showing higher expression in the non-recurrence group compared with the recurrence group in PostNAC samples. Additional File 6. List of Gene Ontology biological processes (GO-BP) and KEGG pathways enriched among genes showing higher expression in the recurrence group compared with the non-recurrence group in PostNAC samples. Additional File 8. List of Gene Ontology (GO) terms and KEGG pathways enriched in the non-recurrence group of PostNAC samples identified by gene set enrichment analysis (GSEA). Enriched gene sets were defined based on normalized enrichment score (NES) and false discovery rate (FDR) q-values. Additional File 9. List of Gene Ontology (GO) terms and KEGG pathways enriched in the recurrence group of PostNAC samples identified by gene set enrichment analysis (GSEA). Enriched gene sets were defined based on normalized enrichment score (NES) and false discovery rate (FDR) q-values. Additional File 10. Clustering and gene expression analysis by RNAseqChef in PreNAC and PostNAC in the recurrence and non-recurrence groups. Additional File 11. Summary of Gene Ontology biological processes (GO-BP) and KEGG pathway enrichment analysis of genes classified into Groups 3 and 4 by RNAseqChef, representing genes upregulated in PostNAC samples compared with PreNAC samples in the recurrence group. Functional enrichment analysis was performed using DAVID. Additional File 12. Summary of Gene Ontology biological processes (GO-BP) and KEGG pathway enrichment analysis of genes classified into Groups 7 and 8 by RNAseqChef, representing genes downregulated in PostNAC samples compared with PreNAC samples in the recurrence group. Functional enrichment analysis was performed using DAVID. Additional File 13. ClinVar-based pathogenic variants in the PostNAC specimen. Additional File 14. ClinVar-based pathogenic variants that occurred or disappeared after NAC.

Supplementary Material 2. Additional File 4. RNAseq of esophageal cancer specimens after neoadjuvant chemotherapy. (a) Summary of biological processes in gene ontology (GO-BP) and KEGG-pathway analysis of genes with elevated expression in non-recurrence. Additional File 7. GSEA based on expression analysis of esophageal cancer samples after neoadjuvant chemotherapy. (a-b) Summary of GO and KEGG analysis of enriched genes in the non-recurrence group by GSEA. NES, normalized enrichment score; GO, gene ontology; KEGG, Kyoto Encyclopedia of Genes and Genomes; GSEA, gene set enrichment analysis.

## Data Availability

Raw sequences were deposited in DDBJ (https://www.ddbj.nig.ac.jp/dra/index-e.html), and the accession numbers were PRJDB37668 and PRJDB37669. However, the above correspondence table linking patient identification codes to personal information is not publicly available due to privacy and ethical constraints. When an application for secondary use of sequence data is submitted, we will ask the applicant to present the purpose of use and review the pros and cons of granting access before making a decision. The person who handles applications for use is Masataka Hirasaki (hirasaki@saitama-med.ac.jp).

## Abbreviations

CAT: Catalase
CF: Cisplatin plus 5-fluorouracil
DEGs: Differentially expressed genes
EC: Esophageal cancer
EMT: Epithelial–mesenchymal transition
ESCC: Esophageal squamous cell carcinoma
FC: Fold change
FDR: False Discovery Rate
FFPE: Formalin-fixed paraffin-embedded
GO: Gene Ontology
GPx: Glutathione peroxidase
GSEA: Gene set enrichment analysis
GSH: Glutathione
GST: Glutathione S-transferase
H&E: Hematoxylin and eosin
ICI: Immune checkpoint inhibitors
INDELs: Insertions/deletions
KEGG: Kyoto Encyclopedia of Genes and Genomes
NAC: Neoadjuvant chemotherapy
OS: Overall survival
PC1: First principal component
PCA: Principal Component Analysis
PostNAC: After CF-based NAC
PPI: Protein–protein interaction
PreNAC: Before CF-based NAC
ROS: Reactive oxygen species
SNVs: Single-nucleotide variants
SOD: Superoxide dismutase
TPM: Transcripts per million
