# Supplementary figures and images for: Glutathione metabolism–associated resistance to cisplatin and 5-FU in esophageal cancer: a paired transcriptomic study
Source: BMC Gastroenterol. 2026 Feb 10;26:169. doi: 10.1186/s12876-026-04664-1 (PMC12990582; doi:10.1186/s12876-026-04664-1)

## Slide 1
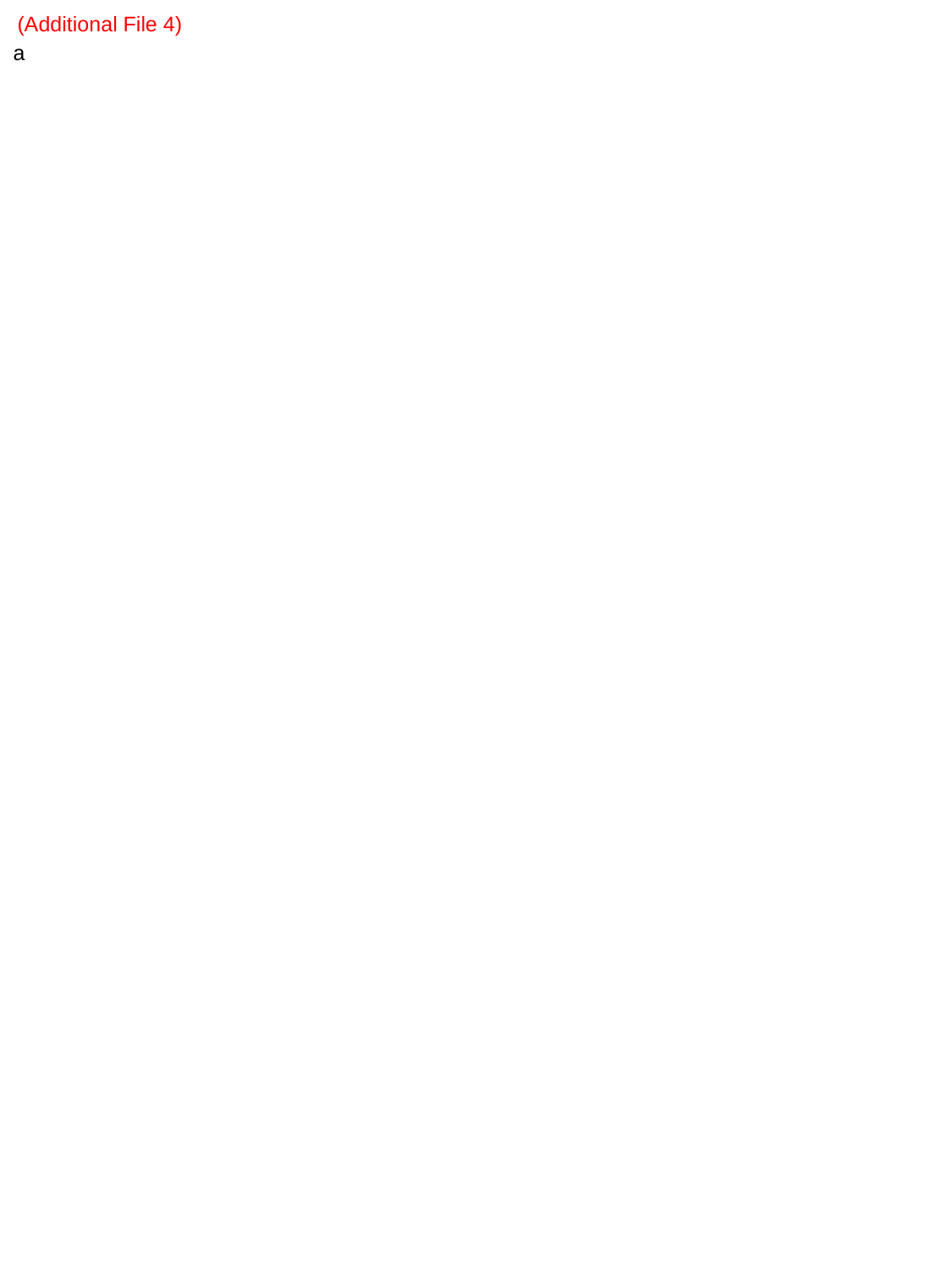

(Additional File 4)
a

## Slide 2
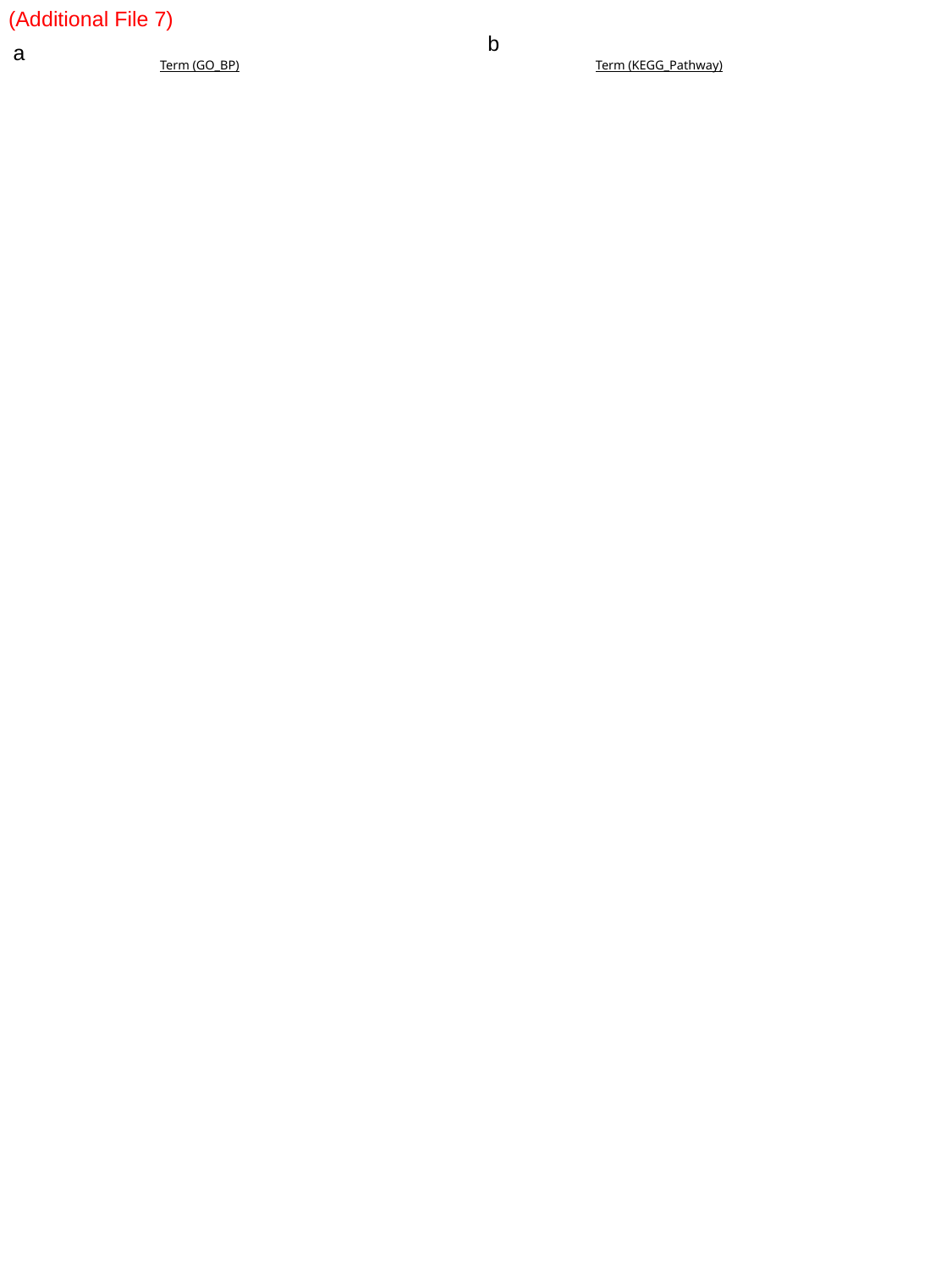

(Additional File 7)
b
a
Term (GO_BP)
Term (KEGG_Pathway)

Supplement: Supplementary file 2 — Supplementary Material 2. Additional File 4. RNAseq of esophageal cancer specimens after neoadjuvant chemotherapy. (a) Summary of biological processes in gene ontology (GO-BP) and KEGG-pathway analysis of genes with elevated expression in non-recurrence. Additional File 7. GSEA based on expression analysis of esophageal cancer samples after neoadjuvant chemotherapy. (a-b) Summary of GO and KEGG analysis of enriched genes in the non-recurrence group by GSEA. NES, normalized enrichment score; GO, gene ontology; KEGG, Kyoto Encyclopedia of Genes and Genomes; GSEA, gene set enrichment analysis. [file 12876_2026_4664_MOESM2_ESM.pptx]
